# Turning tumor inhibition into activation: engineering T cells with chimeric signaling receptors

**DOI:** 10.1186/2051-1426-2-S3-P248

**Published:** 2014-11-06

**Authors:** Ramona Schlenker, Matthias Leisegang, Wolfgang Uckert, Elfriede Noessner

**Affiliations:** 1 Institute of Molecular Immunology, Helmholtz Center Munich, German Research Center for Environmental Health, Munich, Germany; 2 Max Delbrück Center for Molecular Medicine, Helmholtz Association, Berlin, Germany; 3 Institute of Biology, Humboldt-University, Berlin, Germany

## 

Poor *in vivo *persistence and loss of function of adoptively transferred T cells in the tumor milieu are known shortcomings of adoptive T cell therapy (ATT). Providing costimulation might help to improve ATT efficiency. However, human CD8 T effector cells are largely CD28 negative and most tumors do not express CD80 or CD86, thus costimulation cannot be provided via CD28 ligation. We propose to facilitate costimulation of CD8 T effector cells in the tumor milieu through engineering of T cells with a chimeric signaling receptor, which can turn tumor mediated inhibition into activation by abrogating inhibitory PD1 signaling with concomitant activation of the costimulatory pathway. Human T cells engineered to express melanoma specific T cell receptors (TCR) plus the chimeric signaling molecule showed higher ERK phosphorylation associated with stronger IL-2 and IFN-γ secretion upon co-culture with PD-L1 positive target cells. T cells expressing a low avidity TCR achieved functional responses comparable to high avidity TCRs when engineered with the chimeric receptor. The chimeric receptor did not only increase cytokine secretion *in vitro*, but importantly, also supported intra-tumoral proliferation of T cells in a humanized mouse melanoma model.

